# Horizontal alveolar bone loss: A periodontal orphan

**DOI:** 10.4103/0972-124X.75914

**Published:** 2010

**Authors:** A. Jayakumar, S. Rohini, A. Naveen, A. Haritha, Krishnanjeneya Reddy

**Affiliations:** *Department of Periodontics, Sri Sai College of Dental Surgery, Vikarabad, Andhra Pradesh, India*

**Keywords:** Horizontal bone loss, orthopantomographs, vertical / angular defects

## Abstract

**Background:**

Attempts to successfully regenerate lost alveolar bone have always been a clinician’s dream. Angular defects, at least, have a fairer chance, but the same cannot be said about horizontal bone loss. The purpose of the present study was to evaluate the prevalence of horizontal alveolar bone loss and vertical bone defects in periodontal patients; and later, to correlate it with the treatment modalities available in the literature for horizontal and vertical bone defects.

**Materials and Methods:**

The study was conducted in two parts. Part I was the radiographic evaluation of 150 orthopantomographs (OPGs) (of patients diagnosed with chronic periodontitis and seeking periodontal care), which were digitized and read using the AutoCAD 2006 software. All the periodontitis-affected teeth were categorized as teeth with vertical defects (if the defect angle was ≤45° and defect depth was ≥3 mm) or as having horizontal bone loss. Part II of the study comprised search of the literature on treatment modalities for horizontal and vertical bone loss in four selected periodontal journals.

**Results:**

Out of the 150 OPGs studied, 54 (36%) OPGs showed one or more vertical defects. Totally, 3,371 teeth were studied, out of which horizontal bone loss was found in 3,107 (92.2%) teeth, and vertical defects were found only in 264 (7.8%) of the teeth, which was statistically significant (*P*<.001). Search of the selected journals revealed 477 papers have addressed the treatment modalities for vertical and horizontal types of bone loss specifically. Out of the 477 papers, 461 (96.3%) have addressed vertical bone loss, and 18 (3.7%) have addressed treatment options for horizontal bone loss. Two papers have addressed both types of bone loss and are included in both categories.

**Conclusion:**

Horizontal bone loss is more prevalent than vertical bone loss but has been sidelined by researchers as very few papers have been published on the subject of regenerative treatment modalities for this type of bone loss. This study should be an impetus for greater attention to an otherwise ubiquitous periodontal challenge.

## INTRODUCTION

The hallmarks of periodontal disease are inflammation and alveolar bone loss. Gingival inflammation can resolve but alveolar bone loss leads to eventual tooth loss. The aphorism to rheumatic fever — ‘it licks the joints but bites the heart’ — holds true also for periodontal disease. Gingival inflammation improves but the affected bone hardly recovers from the bite.

Attempts to regenerate lost alveolar bone have been elusive and such a goal is always the dream of both the periodontists and the patients. To a certain extent, angular bone loss has been restored, albeit in millimeters and fractions thereof. Such regenerative attempts are at great expense, and even then, the results are not unequivocal; with open-flap debridement alone also showing comparable, if not spectacular, results. If such is the case of angular bone loss with well-entrenched trough-like defects only being eligible for regenerative procedure, the fate of horizontal bone loss is less promising; and it is a frustrating experience for a clinician to face a clinical situation with severe horizontal bone loss.

Kotchy and Laky[1] acknowledged the fact when they stated, “there is no evidence in the literature of attempts at regenerating lost alveolar bone supracrestally.” Improvement of alveolar bone level by use of systemic antibiotics,[2] anti-inflammatory drugs,[3] bisphosphonates,[4,5] distraction osteogenesis,[6] rhBMP-2[7] has been attempted but with mixed and sometimes, discouraging results. Attempts to improve horizontal bone level have also been made with DFDBA in particle, strut and laminar forms in combination with GTR[8] ; use of large membranes to cover extensive periodontal defects[9]; adjunctive use of EMP[10]; GTR and coronally anchored flaps[11]; GTR and osseous grafting with Kielbone[12]; supracrestal placement of tricalcium phosphate ceramic-microfibrillar collagen[13]; composite graft in animals[14] ;and space provision by reinforced ePTFE membrane.[15] Sometimes, the graft material, instead of confining to the infrabony defect, is placed supracrestally also, to regenerate lost alveolar bone, and is often covered with bioresorbable membrane.[1,9]

It is a known fact that horizontal alveolar bone loss is seen more often than the vertical bone defects in periodontal patients. However, information about the prevalence of horizontal bone loss and vertical bone defects is limited. The present authors felt that horizontal bone loss, which contributes to major tooth loss, has not received attention that is due to it, and this is a small effort to focus on an otherwise universal problem.

The purpose of the present study was to evaluate the prevalence of horizontal alveolar bone loss and vertical bone defects in periodontal patients and later to correlate it with the treatment modalities described in the literature for horizontal and vertical bone defects.

## MATERIALS AND METHODS

### Study design

The study was conducted in two parts: part I and part II. Part I comprised the radiographic evaluation of orthopantomographs (OPGs) to find the prevalence of horizontal and vertical bone defects. Part II comprised a search of the literature for various treatment modalities available.

#### Part I — Radiographic evaluation

A total of 150 OPGs from periodontal practice of one of the authors were studied to assess the type of bone loss. Among these 150 OPGs, teeth were excluded if any proximal overlapping was present or if any crowding of teeth was present as it would obscure the CEJ location. All the third molars and normal teeth without periodontitis were excluded from the study. Teeth were also excluded in the anterior area if they were technically blurred and superimposed by natural anatomical landmarks. A total of 677 teeth were rejected for the above reasons, and 152 teeth were missing in the OPGs. Finally, a total of 3,371 teeth were included in the study.

All the OPGs were digitized and loaded into the AutoCAD 2006 software. All the radiographs were examined by one examiner (RS). If this examiner was in doubt about any reference points, a second examiner was also consulted, and the radiographs were included or excluded by consensus. Both the examiners were calibrated, and a pilot study settled some issues pertaining to selection or rejection of a particular radiograph, assessment of extent of bone loss and assignment to either horizontal or vertical bone loss group.

**Figure 1:**
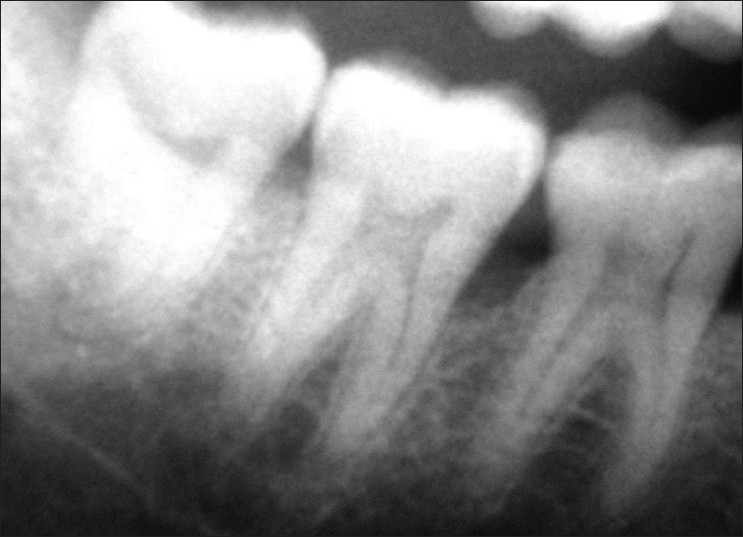
Tooth no. 47 is assigned as being the tooth with vertical bone loss and tooth no. 46, horizontal bone loss

Measurements were made on both mesial and distal surfaces of each tooth. If both the surfaces had horizontal type of bone loss, then the tooth was assigned to have horizontal bone loss. If one of the tooth surfaces had a vertical defect or both the surfaces had vertical defects, then the tooth was assigned to have vertical bone loss. In case of vertical defects, the vertical defect was considered to belong to the tooth along whose root surface the bottom of the defect was present. For example, in the situation depicted in Figure 1, 47 is the tooth assigned to have vertical bone loss; and 46, the tooth to have horizontal bone loss.

**Figure 2:**
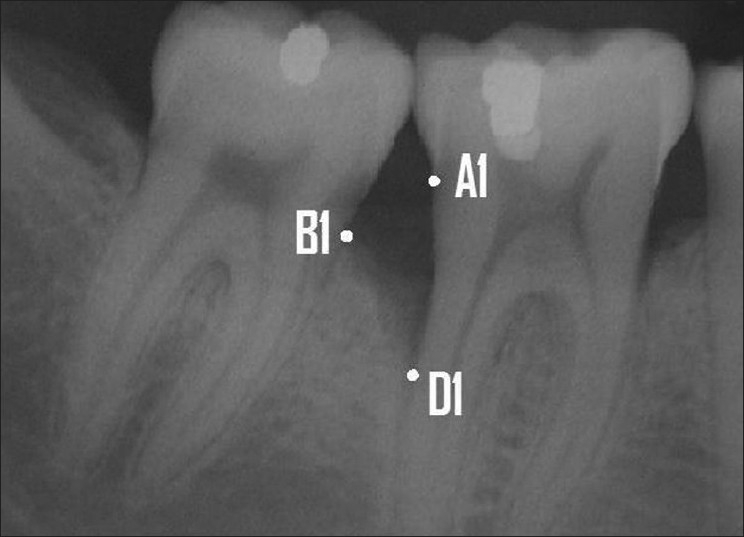
Landmarks selected: A1- CEJ of the tooth involved in the intrabony defect. B1- The most coronal position of the alveolar bone crest of the intrabony defect when it touches the root surface of the adjacent tooth (the top of the crest). D1- The most apical extension of the intrabony destruction where the periodontal ligament still retained its normal width (the bottom of the defect)

A line was drawn from the CEJ to a point where the periodontal ligament space still retained its normal width. A tooth was considered as a periodontitis-affected tooth if the distance was >1.9 mm.[16] A tooth was considered to have a vertical defect amenable for regeneration when the defect angle was ≤45°[17,18] and the intrabony defect depth was ≥3 mm.[19] The radiographic defect angle and width were calculated with the assistance of AutoCAD software. The following anatomical landmarks of the intrabony defect were identified on the digitized radiographs based on the criteria set by Bjorn *et al*.(1969) and Schei *et al*,[20] as shown in Figure 2.

A1: CEJ of the tooth involved in the intrabony defect.

B1: The most coronal position of the alveolar bone crest of the intrabony defect when it touches the root surface of the adjacent tooth (the top of the crest).

D1: The most apical extension of the intrabony destruction where the periodontal ligament still retained its normal width (the bottom of the defect).

**Figure 3:**
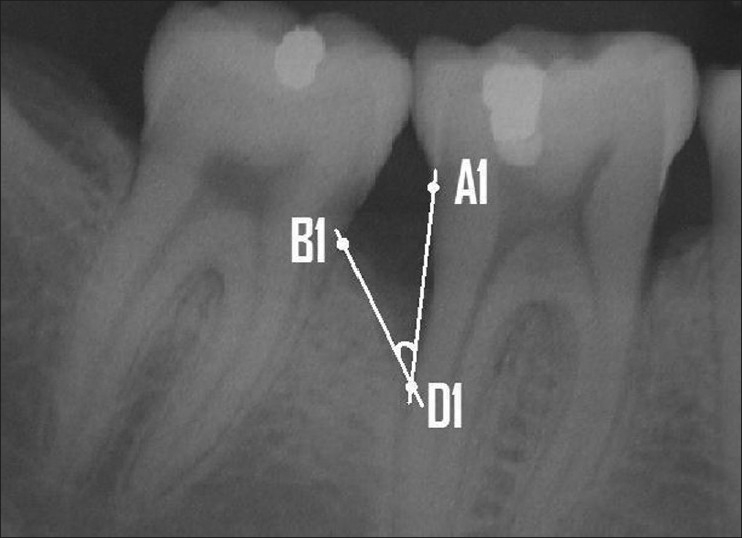
Defect angle was calculated by two lines — one representing the root surface (A1D1) and the other, the bone defect surface (B1D1)

**Figure 4:**
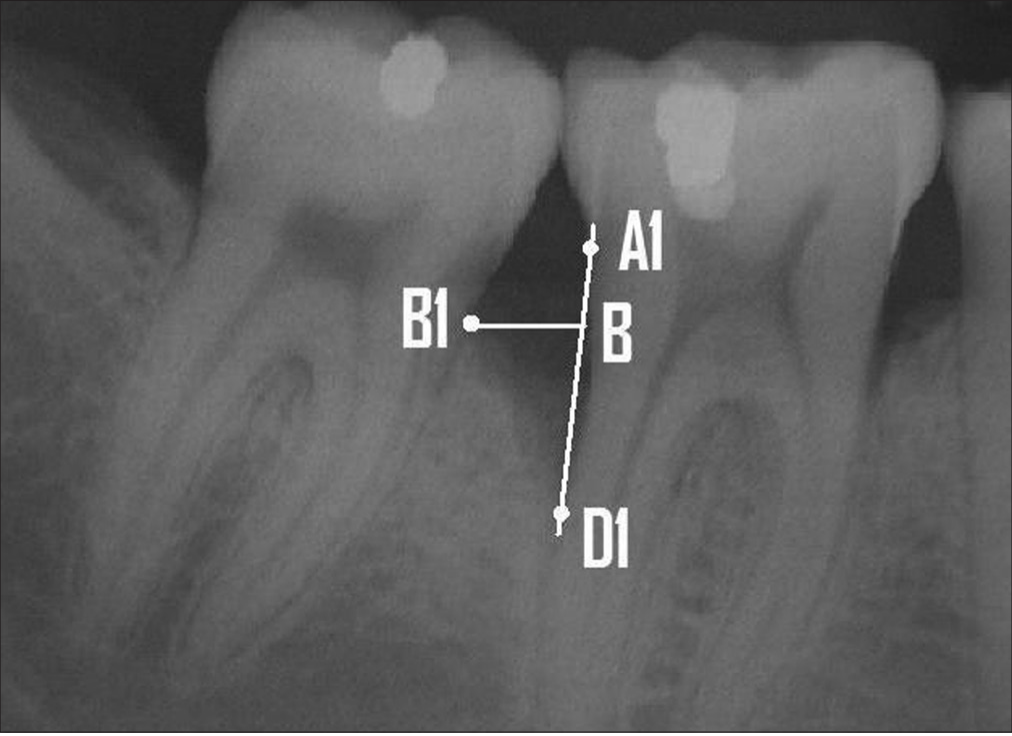
Defect depth was calculated by drawing a horizontal line from B1 to the linear A1D1 line and A1D1 – A1B = BD1 was considered as the defect depth

If restorations were present, the apical margin of the restoration was used to replace the CEJ as a fixed reference point. The radiographic defect angle was then defined by the two lines that represent the root surface (A1D1) and the bone defect surface (B1D1), as described by Steffenson, Weber[17] and Tonetti *et al*.[21] The radiographic intrabony defect was calculated by drawing a horizontal line from B1 to the linear A1D1 line. The point where B1 and A1D1 met was denoted as B and A1D1 – A1B = BD1 was considered as the defect depth. The defect angle and depth are illustrated in Figures 3 and 4.

Thus, with the help of these measurements, the teeth were assigned either as having horizontal bone loss or vertical bone loss.

#### Part II — Literature search

A search of literature for treatment modalities for horizontal and vertical bone loss was conducted in four periodontal journals given in Table 1. One examiner (JK) searched the literature pertaining to treatment options for either horizontal or vertical bone loss. The search was confined to four peer journals of periodontology and for varying periods of their publication. Although there are other journals related to periodontology, they are in existence for relatively shorter periods. Treatment options in other national journals were also considered and dropped because of nonuniformity of periods and also because such cluster of information would not naturally reflect a better picture than that available in journals devoted to periodontal practice alone. The selection of journals and the relevant period was done by consensus among all the authors.

**Table 1:** Journals and their respective publication periods for which they were searched for treatment modalities

Journal name	Publication period - years
International Journal of Periodontics and Restorative Dentistry	1985 - June 2008
Journal of Clinical Periodontology	1985 - June 2008
Journal of Periodontal Research	1985 - June 2008
Journal of Periodontology	1985 - June 2008

The next issue was to consider what type of treatment was considered as pertaining to horizontal bone loss and what was relevant to vertical bone loss. It was easy to pick up published work on treatment options for vertical bone loss as most papers included such nature of defect in the title itself or in the preamble to the paper. Papers which mentioned treatment options for horizontal or supracrestal defects were included under the category of horizontal defect treatment. Both human and animal studies were included for either group.

The articles were hand searched in each journal and assigned to either group by one examiner (JK). A second examiner searched once again in the same fashion; and whenever there was a difference, it was resolved by a conference with other authors and a consensus arrived at accordingly.

### Statistical analysis

Statistical analysis was done using chi-square test. The row percentages and column percentages were calculated and subjected to statistical analysis. There was an association between horizontal and vertical defects at *P*<.001 level. The results, along with statistical analysis, are shown in Table 2

**Table 2:** Distribution of defects in molars, premolars and anteriors

150 OPGs	Molars	Premolars	Anteriors	Total
Horizontal (*n*)	875	865	1367	3107
Row (%)	28.2	27.8	44.0	100
Column (%)	86.2	93.2	95.8	92.2
Vertical (*n*)	141	63	60	264
Row (%)	53.4	23.9	22.7	100
Column (%)	13.8	6.8	4.2	7.8
Total (*n*)	1061	928	1427	3371
Row (%)	30.2	27.5	42.3	
Column (%)	100	100	100	

*P*<.001

## RESULTS

### Part I

Out of the 150 OPGs, 54 (36%) OPGs showed one or more vertical defects ≤45°and ≥3 mm. Out of the 3,371 teeth studied, 3,107 (92.2%) teeth presented horizontal type of bone loss, and only 264 (7.8%) teeth had vertical bone defects which could be considered for regeneration. Out of the 264 vertical defects, majority of the defects were present mesially (145), followed by distal defects (85), and a few teeth had both mesial and distal vertical defects (34). This is shown in Table 3. Molars showed a higher prevalence of vertical defects, followed by premolars, and these defects were least observed in relation to anterior teeth. The observation of least vertical defects in anterior teeth is not unexpected when one considers the fact that the interdental bone between anterior teeth is less voluminous and a vertical bone defect is less prone to occur for anatomical reasons. The results are shown in Table 2.

**Table 3:** Distribution of vertical defects on tooth surfaces

Total vertical defects	Mesial (%)	Distal (%)	Both (%)
264	145 (54.9)	85 (32.2)	34 (12.9)

### Part II

A total of 477 papers addressed the problem of treating horizontal and vertical bone defects, out of which, treatment modalities for vertical bone defects took a lion’s share with 461 papers. A variety of treatment modalities ranging from bone grafts, guided tissue regeneration, root conditioning, enamel matrix derivatives, orthodontic closure of the intrabony defects to a combination of the above procedures were suggested for vertical defects. The horizontal type of bone loss was addressed meagerly with only 18 papers on the subject, while 2 papers addressed both vertical and horizontal defects and hence were placed under both the categories. The results are shown in Table 4.

**Table 4:** Journals with number of research papers dwelling on treatment modalities during the publication years under study

Journal	Vertical defects	Horizontal defects
Journal of Periodontology	183	6
Journal of Clinical Periodontology	134	8
Journal of Periodontal Research	43	0
International Journal of Periodontics and Restorative Dentistry	101	4
Total	461	18
	(96.3%)	(3.7%)

## DISCUSSION

Persson *et al*.[18] have done a prevalence study of horizontal and vertical bone defects. They studied full-mouth, intraoral, periapical radiographs of 416 patients. A total of 10,282 teeth were studied. They stated that in 39.3% (163) of patients, no vertical bone defects were found; and in 30.2% (126) of patients, 3-mm vertical defects were found. In the present study also, 36% of the OPGs (i.e., patients) showed vertical bone loss. The results of the present study that more mesial defects were observed than distal ones and that molars showed more vertical defects than other teeth are in consonance with those of the Persson’s study. The results of the present study are also in agreement with those of the work of Papapanou,[22] who reported 8% of angular defects in the teeth they examined. However, Papapanou presented a different picture regarding the location of defects, asserting that premolars appeared to have a higher prevalence of vertical defects than other teeth.

**Figure 5:**
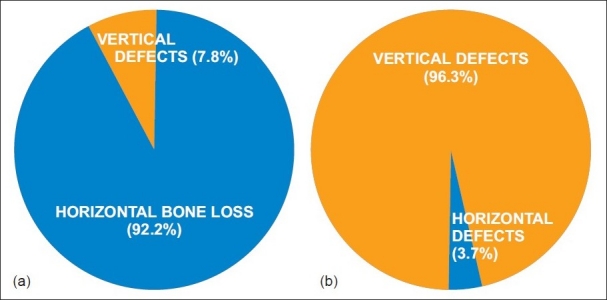
(a) Prevalence of horizontal and vertical defects (b) available treatment options for horizontal defects and vertical defects

It is paradoxical that vertical bone loss, with a prevalence of 7.8%, takes the cake by having 96.8% treatment options; whereas horizontal bone loss, with an overwhelming prevalence of 92.2%, gets scant attention with 3.2% treatment modalities. [Figures 5a and b]

The limitation of the present study is the inherent drawbacks of using OPGs, such as distortion especially in the anterior region. Accuracy of measurements could not be relied upon utilizing OPGs. An ideal method would have been to use intraoral periapical radiographs with paralleling technique. Since this was a retrospective study, the material that was available had to be used. Another drawback of using radiographs to assess the type of bone loss is that, radiographs are two-dimensional images and defects present on buccal and lingual surfaces cannot be identified on OPGs. In fact, visualization of buccal or palatal bone loss is not possible with either intraoral periapical radiographs (IOPAs) or OPGs. Alveolar bone loss does occur to a considerable degree on these surfaces, and the results of this study would have been different had this factor also been taken into consideration.

## CONCLUSION

Horizontal bone loss is the most common problem confronting the clinician but has been receiving scant attention. Research should be directed towards regeneration of lost horizontal bone. Novel technologies using tissue engineering (growth factors[23] and stem cells), miniature bone pins or distraction osteogenesis may emerge as treatment options in the future.
